# The Treatment of Lupus

**Published:** 1884-09

**Authors:** 


### The Treatment of Lupus.

Believing the lupus is just as much a manifestation of scrofula as phthisis, or bone or gland disease, Mr. J. W. Taylor (*Birmingham Med. Review*) makes certain suggestions in accordance with that belief for the treatment of this disorder. The residence should be in a warm and dry climate, the house should be well ventilated, but free from draughts, out-door exercise should never be taken when the weather is cold and wet. The food should be good and varied. Cod-liver oil, when it can be taken, is strongly recommended, and Parrish's food has proved very useful. For local treatment Mr. Taylor recommends scraping with the curette in preference to caustics or scarification. It is best to attack a small part at a time, and remove the disease thoroughly; this treatment is best adapted to those cases where the new growth and ulceration proceed equally; when the latter is in excess, the disease generally gets well without any local remedy; when the former is in excess, free removal is the best treatment.—*London Medical Times.*

